# Correlation between Urine N-Terminal Telopeptide and Fourier Transform Infrared Spectroscopy Parameters: A Preliminary Study

**DOI:** 10.1155/2020/5725086

**Published:** 2020-02-11

**Authors:** Ichiro Okano, Stephan N. Salzmann, Courtney Ortiz Miller, Colleen Rentenberger, Paul Schadler, Oliver C. Sax, Jennifer Shue, Andrew A. Sama, Frank P. Cammisa, Federico P. Girardi, Alexander P. Hughes

**Affiliations:** Spine Care Institute, Hospital for Special Surgery, 535 East 70th St., New York 10021, NY, USA

## Abstract

N-terminal telopeptide (NTX) is a bone resorption marker that is commonly referenced in clinical practice. Bone remodeling is also associated with changes in mineral components. Fourier transform infrared spectroscopy (FTIR) is utilized in the assessment of bone material properties and some parameters are reported to have associations with bone remodeling. The aim of this cross-sectional study is to investigate the relationship between uNTX levels and FTIR parameters, utilizing prospectively collected study data for patients who underwent lumbar fusion surgery. Bone specimens were taken from iliac crest (IC) and vertebrae (V). Cortical (C) and trabecular (T) bones were separately analyzed. 22 patients (mean age 60.0 years (35.9–73.3), male : female 9 : 13) were included in the final analysis. Women showed significantly higher uNTX levels (male : female, median [range] 21.0 [11.0–39.0] : 36.0 [15.0–74.0] nM·BCE/mM, *p*=0.033). Among women, a significant positive correlation was observed between uNTX and mineral-to-matrix ratio in IC-C. Among men, uNTX demonstrated significant negative correlation with collagen crosslinks (XLR: ratio of mature to immature collagen crosslinks) in IC-C, V-T, and V-C. In addition, uNTX was positively correlated with acid phosphate substitution (HPO_4_, a parameter of new bone formation) in IC-C, IC-T, and V-C. After age adjustment, HPO_4_ in IC-T and V-C among men showed significant positive associations with uNTX (IC-T: *p*=0.018, *R*^2^ = 0.544; V-C: *p*=0.007, *R*^2^ = 0.672). We found associations between FTIR parameters and uNTX in men, but not in women. The correlations between uNTX and FTIR parameters in men might suggest a better balance of bone breakdown (uNTX) and new bone formation (FTIR parameters: XLR, HPO_4_) than in women.

## 1. Introduction

Bone turnover includes bone formation and bone resorption processes [1]. It has been reported that bone turnover plays an important role in bone strength and fracture risk [2–5]. Currently, several bone turnover marker assays utilizing blood or urine samples are commercially available. Urine N-terminal telopeptide (uNTX) is a collagen I derived bone resorption marker. uNTX has been one of the most commonly referenced markers in clinical practice [6, 7]. The advantage of utilizing uNTX in daily practice is that it is less sensitive to circadian changes and food intake than other bone turnover makers [8]. High uNTX is an indicator of increased bone resorption. Studies have demonstrated that uNTX is a significant predictor of fracture risk in postmenopausal women in addition to other bone resorption markers [5].

Bone tissue remodeling is also associated with changes in tissue mineral components [9]. Fourier transform infrared spectroscopy (FTIR) is a technique that utilizes a spectrometer that is coupled with a light microscope and allows point-by-point mapping of molecular species composition by detecting differences in reactions to light [10]. FTIR has been used to investigate bone material properties, which is considered one of the major components of bone quality. Bone quality is defined as the determinants of bone strength that does not include bone mineral density (BMD). BMD is a measure of bone quantity, which is another component of bone strength. Although bone turnover markers and bone material property assessment by FTIR have been mostly independently developed, there might be linked associations between these two measures. Previous studies showed that certain FTIR parameters are associated with bone turnover [11, 12]. The purpose of this pilot study is to identify the association between a known bone resorption marker, uNTX, and FTIR parameters that are related to bone turnover, utilizing prospectively collected data.

## 2. Materials and Methods

### 2.1. Ethical Aspects

Institutional ethics board approval was obtained for this study (IRB#2014-084). The study was conducted in agreement with the Declaration of Helsinki II. All participants were provided oral and written information about the purpose of this study and procedures. Written informed consent was obtained from all individual participants included in this study.

### 2.2. Patients and Samples

We obtained data from patients who had preoperative uNTX and available FTIR information from their iliac crest and vertebral bone samples. Patients were identified utilizing prospectively collected data for 60 patients who underwent instrumented posterior lumbar fusion surgery at a single academic institution. Considering racial differences and the effect of antiosteoporotic drugs, we included only Caucasian patients who did not receive drug therapy for osteoporosis. Urine NTX was measured utilizing urine samples taken on the day of preoperative office visit along with urine creatinine and routine preoperative workup (Machine model for uNTX analysis). The measurement of uNTX was performed in the institutional chemistry laboratory utilizing an automated immunoassay (Vitros ECi, Ortho Clinical Diagnostics, Rochester, NY).

During surgery, cortical (C) and trabecular (T) bone samples were obtained from the iliac crest (IC) and vertebral (V) bones. Iliac crest specimens were taken along with bone graft at the site beneath the posterior superior iliac spine, utilizing a 15 mm custom-made trephine set (Depuy Synthes, Raynham, MA), consisting of trephine guide wire, centering pin, drill, and pusher. A bicortical bone tissue sample was collected by penetrating the inner and outer cortical surfaces of iliac crest with the trephine. The size of the iliac crest bone specimen was determined by the thickness of iliac crest. Vertebral bone specimens were collected utilizing a customized 5 mm trephine (Depuy Synthes, Raynham, MA), consisting of drill and pusher, from the operated vertebral body in the same trajectory before pedicle screw insertion. The entry was usually at the confluence of the lateral border of the superior articular facet and the line of mid-transverse process. The trephine was inserted approximately 1 cm into the trabecular bone of the pedicle. Each sample was fixed with ethanol and embedded in polymethyl methacrylate (PMMA). The embedded sample was sliced at 1-2 *μ*m thickness with a microtome (Leica SM 2500, Leica, Germany) and then mounted on barium fluoride (BaF_2_) infrared windows (SpectraTek, Hopewell Junction, NY, USA). Cortical bone and trabecular bone from the same sample were embedded in different blocks and analyzed separately.

### 2.3. FTIR Data Acquisition and Processing

FTIR data from 2 *μ*m sections obtained from embedded in PMMA specimens were collected at 6.25 *μ*m^2^/pixel resolution on an infrared imaging system (Perkin-Elmer Spotlight 300, Perkin-Elmer Instruments, Waltham, MA, USA). From each specimen, a blinded examiner randomly chose five separate areas of intact cortical and trabecular bone for scanning at spectral resolutions of 4 cm^−1^ as described in our previous studies [11, 12]. Utilizing image analytic software (ISYS 5.0 Image Analysis Software, Malvern Instruments, Columbia, MD, USA), images were corrected by subtracting the spectral contribution of PMMA. The following FTIR parameters, as described in previous reports [10, 11, 13–15], were calculated using ISYS software: mineral-to-matrix ratio (Min/Mat), which represents bone mineral content (correlated to ash weight), is calculated from the integrated area of phosphate (916 to 1180 cm^−1^)/amide I (1596 to 1712 cm^−1^) peaks; Carbonate-to-mineral ratio (C/P), which reflects the level of carbonate substitution in the hydroxyapatite (HA) crystal, is calculated from the ratio of the integrated area of the n2 carbonate peak (852 to 890 cm^−1^) and that of the phosphate; crystallinity (XST), which is related to mineral crystal size and perfection as determined by X-ray diffraction, is calculated as the 1030/1020 cm^−1^ peak intensity ratio; collagen crosslink (XLR) network maturity (cross-link ratio) is estimated as the intensity ratio of amide I subbands at 1660 and 1690 cm^−1^; acid phosphate substitution (HPO_4_), which represents the substitution of HPO_4_^−^ ions into hydroxyapatite lattice, characteristics of younger mineral, is calculated from 1128/1096 cm^−1^ peak intensity ratio (Table 1).

### 2.4. Other Variables and Correlation Analyses

The correlation between uNTX and all FTIR parameters was analyzed and stratified by sex. As potential confounders, age, body mass index (BMI), and, if available, volumetric bone mineral density (vBMD) measured by quantitative computed tomography (QCT) were analyzed. QCT-vBMD was calculated using a phantom-less method by converting Hounsfield units (HU) to BMD values with quality assurance (QA) phantom data utilizing Mindways QCT Pro Software (Mindways Software, Inc., Austin, TX, USA).

Statistical analysis was conducted utilizing Mann–Whitney *U* test for continuous variables. Because of the low number of samples, determining linearities was difficult; thus we demonstrated both linear Pearson's correlation and Spearman's nonparametric correlation coefficients for univariate analyses. For FTIR parameter categories (Min/Mat, C/P, XLR, HPO_4_, and XLT) that showed statistically significant correlations in at least one of the pairs, age adjustment by linear regression model was conducted by setting each FTIR parameter as the response variable. All statistical analyses were performed utilizing R software (R for 3.1.0 GUI 1.64). Statistical significance was set at *p* value <0.05.

## 3. Results

### 3.1. Characteristics

22 Caucasian patients (9 males and 13 females, median (range) age 60.0 (35.9–73.3)) with FTIR bone formation parameters and preoperative uNTX were included in the analysis. Three female patients had one or more samples which were unsuitable for FTIR measurement. Therefore, we excluded two iliac crest cortical bone samples, two vertebral trabecular bone samples, and one vertebral cortical bone sample from the final analyses. Two patients (one male and two female patients) did not have QCT compatible preoperative CT; thus vBMDs could not be measured in these cases. None of the patients had a history of clinically significant recent fractures or oncologic, renal, or liver disease.

Women showed significantly higher levels of uNTX (male: median (range), 21.0 (11.0–39.0) nmol BCE/mmol·Cr; female: 36.0 (15.0–74.0) nmol BCE/mmol·Cr, *p*=0.033). The patient demographics and uNTX value stratified by sex are shown in Table 2. No statistically significant differences between male and female patients were observed regarding FTIR parameters (Table 3).

### 3.2. Correlations between uNTX and FTIR Parameters

**Figure 1:**
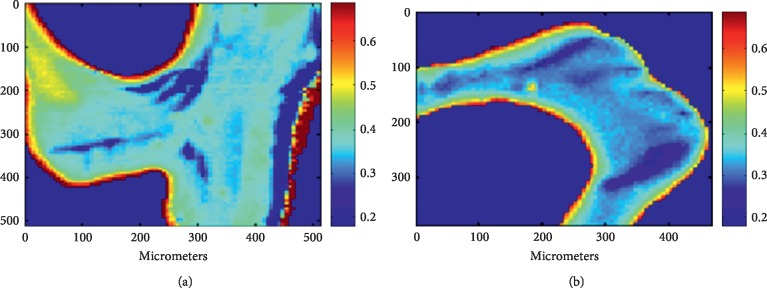
The FTIR images of acid phosphate substitution in iliac crest trabecular bone. All numbers represent scales of micrometer. (a) Patients with high uNTX (39 nmol BCE/mmol·Cr). (b) Patients with low uNTX (14 nmol BCE/mmol·Cr).

In univariate analyses, Min/Mat in IC-C bone showed significance among women in nonparametric Spearman's correlation analysis. No significant correlation was observed between uNTX and other FTIR parameters. Among men, uNTX demonstrated significant negative correlation with XLR in IC-C, V-C, and V-C. In addition, uNTX was positively correlated with HPO_4_ in IC-T, IC-C, and V-C (Table 4 and Figure 1).

Multivariate analyses with age adjustment were conducted for Min/Mat, XLR, and HPO_4_. After adjusting for age, HPO_4_ in IC-T and V-T showed significant positive associations (IC-T: *p*=0.018, *R*^2^ = 0.544; V-C: *p*=0.007, *R*^2^ = 0.672). (Table 5).

## 4. Discussion

Our results demonstrated sexual differences in the associations between FTIR parameters and uNTX. In univariate analyses, uNTX had negative correlations with XLR and positive correlations with HPO_4_ in men. A significant positive nonlinear correlation between Min/Mat and uNTX was observed among women. Since most of the FTIR parameters were associated with age [9, 16], we conducted multivariate analyses with age adjustment. When adjusting for age, only HPO_4_ in iliac trabecular bone and vertebral cortical bone among men showed significant positive associations.

Previous studies demonstrated that the locations of high acid phosphate substitution indicate areas of new bone formation [17, 18]. It was also reported that patients with histomorphometrically defined high-turnover osteoporosis, albeit not statistically significant, demonstrated higher HPO_4_ contents than normal and low-turnover osteoporosis patients [19]. In the present study, there was a significant negative correlation between XLR and uNTX in univariate analyses and also a nonsignificant trend in multivariate analyses. XLR is the ratio of mature-to-immature collagen crosslinks. In age-adjusted patient cohorts, a high XLR reflects aged bone tissue material with greater collagen maturity, which has been associated with fracture risk [13]. Low XLR indicates new bone formation, similar to high HPO_4_.

The balance between bone formation and bone resorption is a primary determinant of bone strength. Osteoporosis develops when the rate of bone resorption exceeds that of bone formation, which leads to a decrease in BMD and the deterioration of bone structure and strength [20]. In the equivalent state, in which bone resorption and formation are balanced, positive correlations between bone resorption markers (uNTX) and FTIR parameters that are related to bone formation (HPO_4_ and XLR), are observed. However, this correlation was only seen among men. In their report about osteoporosis in men, Szulc et al. demonstrated that high bone turnover had a smaller effect on bone mineral density among men and was not an independent risk factor for osteoporotic fractures compared to women [21], whereas there is plenty of evidence that indicates that bone turnover markers can predict osteoporotic fractures in postmenopausal [22] and perimenopausal women [23, 24]. The results of recent studies suggest that the balance between bone resorption and bone formation is disrupted in a considerable proportion of peri- and postmenopausal women. Shieh et al. created a “bone balance index (BBI)” by estimating the relationship between resorption (uNTX) and formation (osteocalcin) markers and showed that a BBI decrease (negative bone balance) was significantly associated with faster BMD decline [23]. Furthermore, Gossiel et al. demonstrated that postmenopausal bone loss in women is associated with both an increase in bone turnover and a negative bone balance [25]. In contrast to women, there are no studies on bone balance issues in men. However, this smaller impact of bone turnover markers on fracture risk in men could be partially explained by our findings, which showed a relatively balanced bone formation in male patients.

Other FTIR parameters, such as Min/Mat, C/P, and XLR, increase when bone tissue is aged or matured [9]. Among these parameters, our results showed that only Min/Mat in iliac crest was significantly positively correlated with uNTX among women in univariate analysis. An increased Min/Mat is a marker for mature or old bone tissue [9]. Previous reports demonstrated that Min/Mat of high bone turnover patients was lower than that of normal subjects [26] and patients undergoing alendronate therapy, which makes uNTX lower, had higher Min/Mat values [27]. According to these results, uNTX should have a negative correlation with Min/Mat, but our univariate analysis demonstrated an inverse result. We believe these inconsistent results might be explained by the effect of age. In general, bone resorption markers increase in peri-/postmenopausal women with advanced age. In fact, no significant association was observed between Min/Mat and uNTX in our multivariate analysis with age adjustment.

Most of the previous human studies utilized iliac crest bone samples only for FTIR measurement and there are no known studies on vertebral bone. In this study, we biopsied bone tissue from two different bone sites: iliac crest and vertebra. There are reports of regional differences in bone material properties between cortical and trabecular bone [13, 15], as well as in different parts of the same bone (femoral neck) [15]. In contrast to previous studies, however, our results showed similar trends between uNTX and each FTIR parameter for two bone sites (iliac crest or vertebra), as well as bone compartments (cortical or trabecular). It remains unclear which region is the best to evaluate overall bone health or fracture risk. The values from a specific site might reflect the fracture risk of the same site better; however, further studies are needed to elucidate the role and clinical implications of regional and anatomical differences.

Despite the prospective design of this study, our study had several limitations, mainly due to its exploratory nature. The first issue is the small sample size of this pilot study; therefore, it cannot be concluded that no correlation exists between nonsignificant variables and uNTX because of low statistical power. A small sample size was also challenging to control potential confounders for bone health. Second, this study analyzed the association between FTIR parameters and a bone resorption marker (uNTX) that is measured in daily practice and the standard bone formation marker (serum procollagen type I N-terminal propeptide (PINP)) was not included. Third, although uNTX is less sensitive to circadian changes and food intake than other bone turnover makers and widely used in daily practice, the official recommendation of the International Osteoporotic Foundation and International Federation of Clinical Chemistry for the reference bone resorption marker in clinical studies is not uNTX, but serum C-terminal telopeptides of type 1 collagen (CTX) instead. The recommendation to use CTX as a reference marker is for facilitating comparison of results among different studies [28]. Thus, care should be taken when comparing the results of this study with others. Moreover, although standard biopsy instructions were provided to operating surgeons, each biopsy sample was subject to differences in manual extraction technique that may not have resulted in specimens from the exact same location. Lastly, samples included for analyses were from Caucasian patients who underwent instrumented posterior lumbar fusion surgery; thus our results might not be generalizable to other patient populations. To overcome these limitations, we are currently performing a prospective study with a larger sample size and refined methodology.

## 5. Conclusion

This study demonstrated an association between a commonly used bone turnover marker uNTX and bone material properties. It also suggests that previously observed sexual differences regarding bone health might be caused by differences in the balance between bone breakdown and new bone formation. These findings might help direct future studies to clarify the underlying mechanism of observed sexual differences in osteoporosis.

## Figures and Tables

**Table 1:** Summary of FTIR parameters.

Parameter	Abbreviation	Definition	Validation
Mineral-to-matrix ratio	Min/Mat	Area of phosphate (916 to 1180 cm^−1^) band/area of amide I (1596 to 1712 cm^−1^) band	Bone mineral content, correlated to ash weight

Carbonate-to-phosphate ratio	C/P	Area of the carbonate (852 to 890 cm^−1^) band/area of the phosphate (916 to 1180 cm^−1^) band	The level of carbonate substitution in the hydroxyapatite (HA) crystal

Crystallinity	XST	Intensity ratio of subbands at 1030/1020 cm^−1^	Mineral crystal size and perfection as determined by X-ray diffraction

Collagen cross link	XLR	Intensity ratio of amide I subbands at 1660/1690 cm^−1^	Collagen cross-link network maturity, ratio of mature and immature collagen crosslinks

Acid phosphate substitution	HPO_4_	Intensity ratio of subband at 1128/1096 cm^−1^	The substitution of HPO_4_^−^ ions into hydroxyapatite lattice, characteristics of younger mineral

**Table 2:** Patient demographics.

Factor		Overall	Male	Female	*p* value
*N*		22	9	13	
Age	Median (range)	60.02 [35.96, 73.25]	60.20 [35.96, 71.00]	59.85 [42.13, 73.25]	0.556
BMI (Kg/m2)	Median (range)	28.79 [20.12, 42.45]	31.66 [25.40, 42.45]	26.60 [20.12, 37.79]	0.071
L1.2 vBMD (mg/cm3)	Median (range)	118.95 [71.65, 186.45]	126.03 [94.30, 170.40]	118.95 [71.65, 186.45]	0.545
uNTX (nmol BCE/mmol·Cr)	Median (range)	29.50 [11.00, 74.00]	21.00 [11.00, 39.00]	36.00 [15.00, 74.00]	0.033

**Table 3:** Distribution of FTIR parameters.

Factor		Overall	Male	Female	*p* value
*N*		22	9	13	
Min/Mat	Iliac trabecular	4.27 [4.00, 5.32]	4.24 [4.10, 5.32]	4.36 [4.00, 4.72]	0.664
	Iliac cortical	4.50 [3.88, 6.26]	4.48 [3.88, 6.26]	4.60 [4.28, 5.42]	0.305
	Vertebral cortical	4.52 [3.96, 5.30]	4.93 [3.96, 5.30]	4.34 [4.06, 4.84]	0.112
	Vertebral trabecular	4.50 [3.84, 5.76]	4.72 [3.86, 5.76]	4.42 [3.84, 4.76]	0.064

C/P	Iliac trabecular	0.0082 [0.0055, 0.0096]	0.0081[0.0055, 0.0096]	0.0082 [0.0073, 0.0092]	0.893
	Iliac cortical	0.00835 [0.0074, 0.0092]	0.0081 [0.0077, 0.0092]	0.0084 [0.0074, 0.0091]	0.567
	Vertebral trabecular	0.0079 [0.0051, 0.0100]	0.0080 [0.0051, 0.0089]	0.00785 [0.0068, 0.0100]	0.776
	Vertebral cortical	0.00855 [0.00530, 0.0105]	0.0087 [0.0053, 0.0099]	0.0083 [0.0074, 0.0105]	0.647

XLR	Iliac trabecular	3.92 [3.16, 4.64]	3.98 [3.28, 4.64]	3.92 [3.16, 4.58]	0.738
	Iliac cortical	4.05 [3.14, 4.86]	4.14 [3.40, 4.86]	4.00 [3.14, 4.38]	0.704
	Vertebral trabecular	4.34 [3.24, 5.40]	4.26 [3.52, 5.40]	4.36 [3.24, 5.12]	0.859
	Vertebral cortical	4.28 [3.28, 5.00]	4.44 [3.30, 5.00]	4.24 [3.28, 4.62]	0.676

HPO_4_	Iliac trabecular	0.37 [0.34, 0.45]	0.37 [0.34, 0.40]	0.37 [0.34, 0.45]	0.640
	Iliac cortical	0.34 [0.28, 0.41]	0.34 [0.28, 0.36]	0.34 [0.30, 0.41]	0.819
	Vertebral trabecular	0.38 [0.31, 0.47]	0.38 [0.31, 0.41]	0.38 [0.33, 0.47]	0.702
	Vertebral cortical	0.35 [0.30, 0.40]	0.36 [0.30, 0.40]	0.34 [0.33, 0.38]	0.941

XST	Iliac trabecular	1.22 [1.19, 1.24]	1.21 [1.19, 1.24]	1.22 [1.19, 1.24]	0.616
	Iliac cortical	1.22 [1.16, 1.25]	1.22 [1.19, 1.25]	1.24 [1.16, 1.25]	0.470
	Vertebral trabecular	1.21 [1.14, 1.26]	1.19 [1.14, 1.26]	1.22 [1.16, 1.26]	0.422
	Vertebral cortical	1.22 [1.16, 1.27]	1.20 [1.16, 1.25]	1.23 [1.20, 1.27]	0.056

**Table 4:** Correlations between uNTX and FTIR parameters: univariate analyses.

		Male	*n* = 9			Female	*n* = 13		*p* value
		*r*	*p* value	*ρ*	*p* value	*r*	*p* value	*ρ*	
Age		−0.226	0.559	−0.420	0.260	0.027	0.931	0.132	0.667
BMI		−0.219	0.571	−0.277	0.470	−0.116	0.706	−0.399	0.177
L1.2 vBMD		0.165	0.696	0.265	0.526	0.233	0.490	0.305	0.361

Min/Mat	Iliac trabecular	−0.281	0.463	−0.122	0.754	0.511	0.075	0.541	0.056
	Iliac cortical	−0.364	0.335	−0.420	0.260	0.516	0.104	**0.671**	**0.024**
	Vertebral trabecular	0.480	0.191	0.588	0.096	0.158	0.624	0.221	0.491
	Vertebral cortical	0.243	0.530	0.412	0.271	0.183	0.589	0.210	0.536

C/P	Iliac trabecular	0.292	0.446	0.242	0.531	0.211	0.490	0.044	0.886
	Iliac cortical	0.296	0.439	0.123	0.752	0.292	0.384	0.137	0.688
	Vertebral trabecular	0.578	0.103	0.655	0.055	0.306	0.334	0.474	0.120
	Vertebral cortical	0.330	0.386	0.266	0.489	0.008	0.981	−0.069	0.841

XLR	Iliac trabecular	−0.402	0.283	−0.608	0.083	0.151	0.623	0.229	0.452
	Iliac cortical	−**0.672**	**0.047**	−**0.790**	**0.011**	0.158	0.642	0.139	0.683
	Vertebral trabecular	−**0.703**	**0.035**	−**0.681**	**0.044**	0.088	0.786	0.214	0.505
	Vertebral cortical	−**0.671**	**0.048**	−0.605	0.084	0.123	0.719	0.155	0.649

HPO_4_	Iliac trabecular	**0.808**	**0.008**	**0.790**	**0.011**	−0.347	0.246	−0.251	0.409
	Iliac cortical	**0.680**	**0.044**	**0.752**	**0.019**	−0.388	0.239	−0.365	0.269
	Vertebral trabecular	0.516	0.155	0.454	0.220	−0.024	0.940	−0.102	0.753
	Vertebral cortical	**0.865**	**0.003**	**0.790**	**0.011**	−0.021	0.951	0.105	0.759

XST	Iliac trabecular	−0.551	0.124	−0.546	0.128	0.334	0.264	0.331	0.270
	Iliac cortical	−0.124	0.750	−0.042	0.915	0.352	0.288	0.337	0.311
	Vertebral trabecular	−0.451	0.223	−0.521	0.150	−0.382	0.221	−0.560	0.058
	Vertebral cortical	−0.357	0.346	−0.412	0.271	0.100	0.771	−0.164	0.630

Statistically significant variables shown in **bold**.

**Table 5:** The results of multivariate analyses with age adjustment.

		Male	*n* = 9	Adjusted *R*^2^	Female	*n* = 13	Adjusted *R*^2^
		*β*	*p*-value		*β*	*p*-value	
Min/Mat	Iliac trabecular	−0.00825	0.580	NA	0.00716	0.063	0.319
	Iliac cortical	−0.01579	0.481	0.254	0.01024	0.070	0.352
	Vertebral trabecular	0.02964	0.169	0.077	0.00278	0.595	NA
	Vertebral cortical	0.01887	0.218	0.360	0.00300	0.524	NA

XLR	Iliac trabecular	−0.01686	0.316	NA	0.00269	0.630	NA
	Iliac cortical	−0.03270	0.069	0.273	0.00307	0.665	NA
	Vertebral trabecular	−0.03896	0.061	0.389	0.00165	0.858	NA
	Vertebral cortical	−0.03979	0.080	0.280	0.00250	0.738	NA

HPO_4_	Iliac trabecular	**0.00150**	**0.018**	**0.544**	−0.00058	0.268	NA
	Iliac cortical	0.00173	0.073	0.401	−0.00064	0.255	NA
	Vertebral trabecular	0.00139	0.220	0.043	−0.00005	0.943	NA
	Vertebral cortical	**0.00279**	**0.007**	**0.672**	−0.00004	0.903	NA

NA: not applicable (adjusted *R*^2^ values were below null value).

## Data Availability

The clinical and experimental data used to support the findings of this study have not been made available because of the data protection policy of the Hospital for Special Surgery. Further information about the data and conditions for access are available from the corresponding author upon reasonable request.

## Supplementary Materials

Supplementary Materials STROBE Statement—checklist of items that should be included in reports of observational studies.
